# Nur77-Tempo mice reveal T cell steady state antigen recognition

**DOI:** 10.1093/discim/kyac009

**Published:** 2022-12-22

**Authors:** Thomas A. E. Elliot, Emma K. Jennings, David A. J. Lecky, Sophie Rouvray, Gillian M. Mackie, Lisa Scarfe, Lozan Sheriff, Masahiro Ono, Kendle M. Maslowski, David Bending

**Affiliations:** 1 Institute of Immunology and Immunotherapy, College of Medical and Dental Sciences, University of Birmingham, Birmingham B15 2TT, UK; 2 Department of Life Sciences, Imperial College London, London, SW7 2AZ, UK

**Keywords:** TCR signalling, Nur77, antigen, Nr4a1, Tempo

## Abstract

In lymphocytes, *Nr4a* gene expression is specifically regulated by antigen receptor signalling, making them ideal targets for use as distal T cell receptor (TCR) reporters. *Nr4a3*-Timer of cell kinetics and activity (Tocky) mice are a ground-breaking tool to report TCR-driven *Nr4a3* expression using Fluorescent Timer protein (FT). FT undergoes a time-dependent shift in its emission spectrum following translation, allowing for the temporal reporting of transcriptional events. Our recent work suggested that *Nr4a1*/Nur77 may be a more sensitive gene to distal TCR signals compared to *Nr4a3*, so we, therefore, generated Nur77-Timer-rapidly-expressed-in-lymphocytes (Tempo) mice that express FT under the regulation of Nur77. We validated the ability of Nur77-Tempo mice to report TCR and B cell receptor signals and investigated the signals regulating Nur77-FT expression. We found that Nur77-FT was sensitive to low-strength TCR signals, and its brightness was graded in response to TCR signal strength. Nur77-FT detected positive selection signals in the thymus, and analysis of FT expression revealed that positive selection signals are often persistent in nature, with most thymic Treg expressing FT Blue. We found that active TCR signals in the spleen are low frequency, but CD69^+^ lymphoid T cells are enriched for FT Blue^+^ Red^+^ T cells, suggesting frequent TCR signalling. In non-lymphoid tissue, we saw a dissociation of FT protein from CD69 expression, indicating that tissue residency is not associated with tonic TCR signals. Nur77-Tempo mice, therefore, combine the temporal dynamics from the Tocky innovation with increased sensitivity of *Nr4a1* to lower TCR signal strengths.

## Graphical abstract

**Graphical Abstract:**
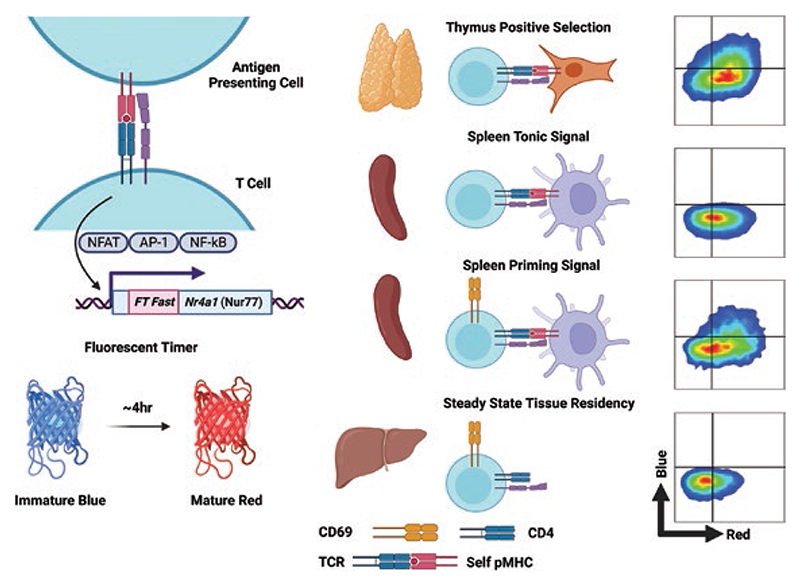

## Introduction

Activation of T cells is tightly controlled by their T cell receptor (TCR). Signalling pathways downstream of the TCR show different dynamics. For example, the calcineurin-Nuclear Factor of Activated T-cells (NFAT) pathway relies on the release of intracellular Ca^2+^ and occurs in a digital ‘all or nothing’ fashion [1]. Similar binary signalling occurs in the Rat Sarcoma Virus/Extracellular Signal Related-Kinase (RAS/ ERK) pathway [2]. In contrast, the magnitude of signalling via Nuclear Factor Kappa B (NF-κB) is graded in relation to TCR signal strength by the activity of Interleukin-2-Inducible Kinase (ITK) [3]. TCR signal strength is a key determinant of T cell function and differentiation. The affinity of the TCR for self-antigen mediates clonal deletion and regulatory T cell (Treg) development in the thymus [4]. Higher affinity for antigen can pre-dispose CD4 and CD8 T cells towards an effector fate over memory [5, 6], and TCR signal strength calibrates negative feedback and subsequent activation threshold [7].

TCR ligation drives the expression of CD69, which binds to sphingosine-1-phosphate receptor 1 (S1PR_1_) mediating its internalisation. Internalisation of S1PR_1_ prevents lymph node egress due to loss of sensitivity to chemoattractant sphingosine-1-phosphate, which is concentrated in the blood [8]. CD69 is downregulated to allow egress to peripheral tissues but can then be re-expressed and maintained in tissue resident T cells to prevent re-circulation into the blood. Tissue resident T cells may then express adhesion molecules CD49a and CD103 that bind to collagen and E-cadherin, respectively, to sustain tissue retention. Here, they can persist for years [9], even after clearance of infection [10]. It has been proposed that peripheral re-encounter with antigen drives re-expression of CD69 [11] to initiate the tissue residency programme; however, there have been reports that tissue resident T cells persist in the absence of antigen and that residency programmes are instead driven by the tissue microenvironment [12–14]. Thus, the role of TCR signalling in the development and maintenance of tissue residency is unclear.

Whilst CD69 is upregulated as a result of TCR signalling, it has also been shown to upregulate in response to type I interferons [15]; therefore, expression of CD69 cannot distinguish between T cells activated by TCR signals and those activated by the inflammatory environment. In humans and mice, early TCR signalling is associated with the expression of the NR4A family of nuclear orphan receptors [16], and their transcripts can be used as biomarkers of TCR signalling. In mice, a GFP reporter of *Nr4a1* (Nur77-GFP) has been used to track TCR signalling events *in vivo* [17]. The reporter was unresponsive to innate stimuli or inflammatory cytokines, demonstrating that *Nr4a1* expression is a specific read-out of TCR signalling. Similar results have been shown in human T cells [18]. The brightness of the Nur77-GFP reporter increases with the affinity and duration of TCR signals [17, 19, 20], demonstrating that the expression level of *Nr4a1* is graded in relation to TCR signal strength.

The Ono laboratory invented the ground-breaking *Nr4a3* Timer of cell kinetics activity (Tocky) system to trace TCR signalling [21]. The system exploited a Fluorescent Timer (FT) protein, which is initially translated into an unstable blue form, then undergoes a time-dependent shift in its emission spectrum into a mature red form with a half-life of ~4 hours [21, 22]. Therefore, the *Nr4a3*-Tocky model can be used to distinguish TCR signalling events that are historic (>~4–7 hours ago; FT Red) or recent (<~4–7 hours ago; FT Blue) [21]. Expression of *Nr4a3* is dependent on NFAT signalling and requires stronger and/or longer TCR signals than *Nr4a1* [19, 23]. In contrast to the graded behaviour of *Nr4a1* expression, antigen-driven expression of *Nr4a3* occurs in a digital fashion [7]. Here, we aimed to utilize both the time resolution of Tocky and the increased sensitivity of *Nr4a1*/Nur77 {henceforth referred to as Nur77-Timer rapidly expressed in lymphocytes [Tempo]} expression over *Nr4a3*. The Nur77-Tempo mouse allowed us to interrogate steady state antigen recognition within the intact mouse.

## Materials and methods

### Mice

*Nr4a3*-Tocky mice were generated in the Ono lab as previously described [21]. Nur77-Tempo mice were generated by Taconic/Cyagen using their ‘Piggybac-on-BAC’ approach. Using bacterial artificial chromosome (BAC) RP24-366J14, which contains the entire *Nr4a1* gene, a Fast-FT-rBG pA Zeomycin (Zao) cassette was inserted upstream of the *Nr4a1* ATG start codon located in exon 2 (the first coding exon). The *Zeo* cassette was deleted by homologous recombination. A pair of piggyBac inverted terminal repeats (ITRs) flanking an ampicillin resistance cassette were introduced into the modified BAC vector backbone. The two ITR elements are recognition sequences of the piggyBac transposase (PBase), which facilitates integration into random TTAA sites by PBases, with one copy per integration site. The Modified BAC was injected into single-cell stage fertilized eggs. Pups were genotyped by PCR to screen for founder lines. A total of eight founder lines were generated, of which four had germline transmission. All animal experiments were approved by the local animal welfare and ethical review body and authorized under the authority of Home Office licenses P18A892E0A and PP3965017 (held by Dr. David Bending). Animals were housed in specific pathogen-free conditions.

### Primers

*Nr4a1-Ft Fast BAC* (product size 362bp): For: GTGTACCC GTCCATGAAGGTGCT, Rev: CTGCTGTCCATTCCTTATTCCATAG
*Il2ra:* For: CAGGAGTTTCCTAAGCAACG Rev: CTG TGTCTGTATGACCCACC
*Ft Fast:* For: CGCGGAACTAACTTCCCCTC Rev: GTC TTGACCTCAGCGTCGTA

### *In vitro* culture

Single-cell suspensions of splenocytes were generated as previously described [19] and were cultured in RPMI containing 10% FBS (v/v) 1% penicillin/streptomycin (v/v) at 37°C 5% CO_2_. B cells were activated with 1 μg/mL anti-IgM (polyclonal). T cells were activated with 1 μg/mL anti-CD3ε (clone 145.2C11) stimulating antibody with or without small molecule inhibitors indicated in the resources table or DMSO vehicle control. Inhibitor concentrations are indicated in figure legends. Following *in vitro* activation, T cells or B cells were analysed by flow cytometry.

### *In vivo* T cell activation

Mice were weighed and then injected intraperitoneally with 1 mg/kg anti-CD3ε (BioLegend, clone 145.2C11). Mice were culled 4 hours later, and spleens were removed for analysis.

### Tissue preparations

Spleen and thymus were prepared as previously described [19]. Lungs were perfused with PBS and minced using a scalpel before 40 mins digest in RPMI 10% FBS (v/v), 1% penicillin–streptomycin (v/v), 1 mg/mL collagenase D (w/v), 1 mg/mL dispase (w/v), 40 μg/mL DNase I (w/v). Digested lung tissue was passed through 100 μM filter and centrifuged at 1500 rpm for 5 mins. Red blood cells were lysed in RBC lysis buffer for 2 mins or ice before washing in PBS. Resulting sample was passed through a 40 μm filter, resuspended in FACS buffer, then analysed by flow cytometry. Livers were perfused with PBS and forced through 70 μM cell strainers. Homogenate was topped up to 15 mL with ice-cold RPMI and centrifuged at 2000 rpm for 5 mins. Resulting pellet was resuspended in 10 mL ice-cold RPMI and layered over OptiPrep density gradient medium (Sigma), then centrifuged at 1000 g for 25 mins at deceleration setting 3. Leukocyte layer was aspirated, washed in RPMI, resuspended in FACS buffer, the analysed by flow cytometry. Lymphocytes from small intestine epithelial layer were isolated as previously described [24].

### Genotyping

**Figure 1:**
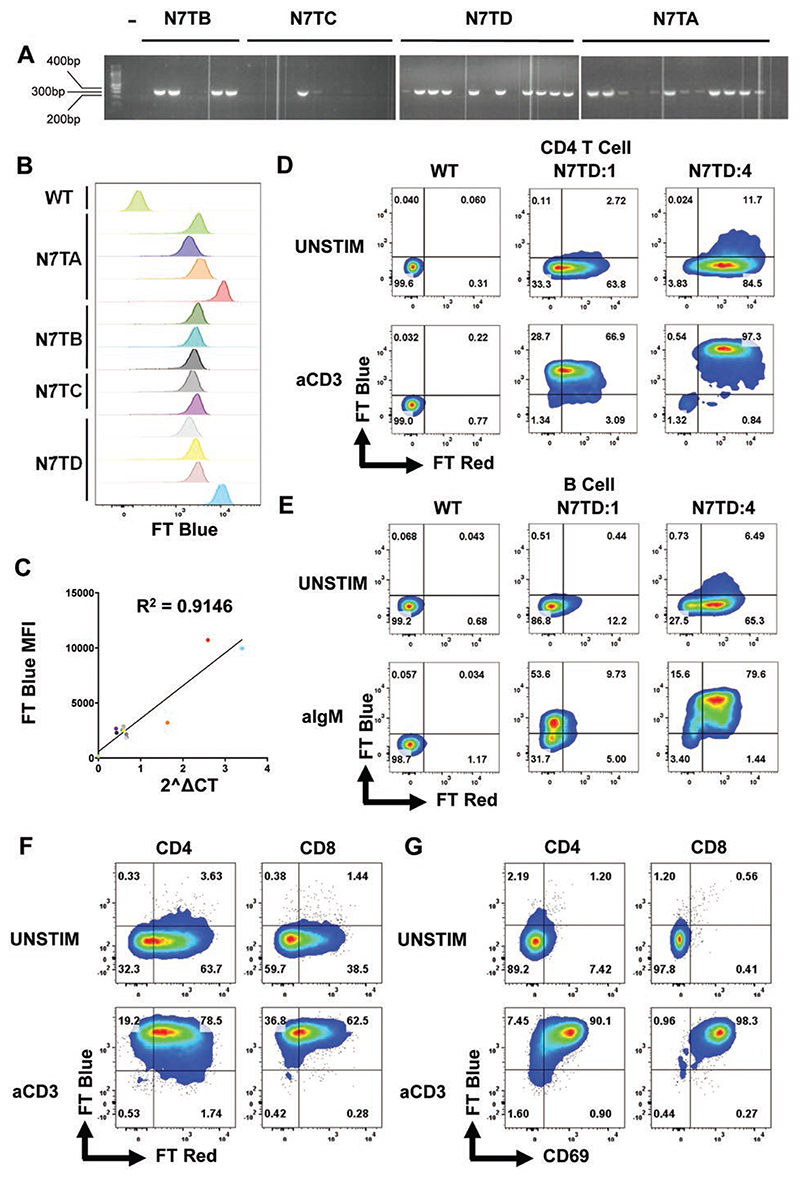
Generation and validation of Nur77-Tempo. (A) *Nur77-Tempo* founder lines (N7TA-D) were mated to C57BL/6 mice. Genomic DNA extracted from ear punch biopsy was subjected to end-point PCR amplification of *Nur77-Ft Fast BAC*. Amplified DNA stained and separated by gel electrophoresis. Image shows three separate gels. (B) Expression of FT Blue analysed in individuals with positive PCR band by flow cytometry following 4-hour *in vitro* stimulation of bulk splenocytes with soluble anti-CD3, gated on live CD4^+^ T cells (Supplementary Fig. 2). (C) Expression of *Ft Fast* quantified by RT–PCR (see Methods) and correlated with the MFI of FT Blue. *R*^2^ value showed is pearson rank correlation coefficient. (D) Expression of FT Blue and FT Red in live CD4^+^ T cells following 4-hour *in vitro* stimulation of bulk splenocytes with 1 μg/mL soluble anti-CD3. Shown in wild-type mice (WT), estimated single copy progeny from founder D (N7TD:1), estimated four copy progeny from founder D (N7TD:4). (E) Expression of FT Blue and FT Red in live CD19^+^ B cells following 4 hours *in vitro* stimulation of bulk splenocytes with 1 μg/mL soluble anti-IgM. Shown in WT, estimated single copy progeny from founder D (N7TD:1), estimated four copy progeny from founder D (N7TD:4). (F) Expression of FT Blue and FT Red in live CD4^+^ and CD8^+^ T cells following 4-hour *in vivo* stimulation by intraperitoneal injection of 1 mg/kg anti-CD3. (G) Expression of FT Blue and CD69 in live CD4^+^ and CD8^+^ T cells following 4-hour *in vivo* stimulation by intraperitoneal injection of 1 mg/kg anti-CD3.

Genomic DNA was extracted from ear punch biopsies using ThermoFisher genomic DNA mini kit. For endpoint PCR, *Ft Fast* genomic DNA was amplified using New England Biolabs OneTaq mastermix according to the manufacturer’s instruction using an annealing temperature of 60°C. Samples were separated by gel electrophoresis and visualized using Invitrogen SYBR safe DNA gel stain. To determine copy number, RT–PCR of genomic DNA was performed using Applied Biosystems PowerUp SYBR green mastermix. Copy number was estimated by {inline figure 1}, based on a presumption that the lowest delta cycle threshold (Ct) value represented a single copy transgenic, and a single copy 2^ΔCT^ averaged ~0.7 (see Fig. 1C).

### Flow cytometry

Cells were stained in 96 well U-bottom plates with an antibody mastermix and fixable viability dye diluted in 2% FBS PBS (v/v) at 4°C for 30 mins in the dark. Cells were washed in 2% FBS PBS (v/v) and then analysed on a BD LSR Fortessa X-20. FT Blue was excited by the 405 nm laser and detected in the 450/40 nm filter, FT Red was excited by the 561 nm laser and detected by the 610/20 nm filter.

### Instruments and software

BD LSR Fortessa X-20 flow cytometer.
ThermoFisher Quant Studio 5 Real Time PCR System
GraphPad Prism 9
Flowjo v10.8.1 (BD Biosciences)

### Statistical analysis

Two-way ANOVA analysis with Sidak’s multiple comparison was employed. Bars represent mean ± SEM unless otherwise stated. Linear regression was performed using Pearson’s rank correlation. *P < 0.05, **P < 0.01, ***P < 0.001, ****P < 0.0001.

**Table:** Reagents table

Target	Supplier	Catalogue ID
CD4 (GK1.5) BUV737	BD Biosciences	564298; RRID: AB_2738734
CD8α (53-6.7) BUV395	BD Biosciences	563786; RRID: AB_2732919
CD8α (53-6.7) FITC	BioLegend	100706; RRID: AB_312744
CD8β (53-5.8) PerCP-Cy5.5	BioLegend	140417; RRID: AB_2800650
TCRβ (H57-597) AF700	BioLegend	109224; RRID: AB_1027648
TCRγδ (GL3) APC	BioLegend	118116; RRID: AB_1731813
CD25 (PC61) PE-Cy7	BioLegend	102016; RRID: AB_312865
CD69 (H1.2F3) FITC	BioLegend	104506; RRID: AB_313109
CD69 (H1.2F3) AF700	BioLegend	104539; RRID: AB_2566304
Anti-IgM (Polyclonal)	BioLegend	157102; RRID: AB_2814087
anti-CD3ε Ultra LEAF (145-2C11)	BioLegend	100301; RRID: AB_312666
Reagent	Supplier	Catalogue ID
eFluor780 Viability dye	eBioscience	65-0865-18
RPMI w/L-Glut	Gibco	21875034
Penicillin/Streptomycin	Gibco	15140122
Fetal Bovine Serum	Gibco	10500064
PBS	Sigma–Aldrich	10500064
Collagenase D	Merck	11088858001
DNase I	Merck	10104159001
Dispase II	Sigma	102405030
RBC lysis buffer	Thermo Fisher	00-4333-57
OptiPrep	Sigma	D1556
DMSO	Sigma	D2650-100ML
PP2	Sigma–Aldrich	P0042-5MG
GO 6983	Cambridge Bioscience	SM20-1
SP600125	Sigma–Aldrich	S5567-10MG
Ly 294002	Promega	V1201
PD0325901	Cambridge Bioscience	SM26-2
Cyclosporin A	Cambridge Bioscience	SM43-50
Genomic DNA Mini Kit	Thermo Fisher	K182001
OneTaq 2X MasterMix	New England Biolabs	M0482
PowerUp SYBR Green Mastermix	Applied Biosystems	A25742
SYBR Safe DNA Gel Stain	Thermo Fisher	S33102

## Results

Transgenic mice were generated using a BAC strategy to express the *Ft Fast* reporter under the regulation of *Nr4a1* (Nur77-Tempo; Supplementary Fig. 1). Founder lines were mated to C57BL/6J mice, and progeny were analysed for expression of the *Ft Fast* transgene by end-point PCR and gel electrophoresis (Fig. 1A). Individuals from all four founder lines (N7TA-D) showed positive bands at expected size of 382 bp, indicating successful insertion of *Ft Fast* and were selected for further screening. Splenocytes from *Ft Fast+* mice were stimulated *in vitro* with anti-CD3 before flow cytometry analysis of FT Blue expression in CD4^+^ T cells (Fig. 1B; gating strategy Supplementary Fig. 2). All individuals expressed FT Blue in response to *in vitro* stimulation, confirming insertion of the transgene into chromatin regions accessible in mature T cells. Some individuals showed brighter expression of FT Blue, indicating elevated transcript number of *Ft Fast*. RT– PCR of genomic DNA was used to estimate copy number; those with multiple copies of *Ft Fast* had a higher median fluorescent intensity (MFI) upon stimulation (Fig. 1C and D), confirming multiple insertion sites resulted in increased brightness of FT Blue.

Comparison of an estimated four copy variant with a single copy variant revealed elevated proportions of CD4^+^ T cells and B cells that express FT Red, demonstrating that multiple insertion sites increase the sensitivity of the reporter for steady state tonic signals in both T and B cells (Fig. 1D and E). FT Blue expression was also observed in B cells stimulated *in vitro* with anti-IgM (Fig. 1E). However, a proportion of B cells in the single copy variant failed to express FT Blue in response to *in vitro* stimulation, and those that did showed comparatively lower brightness compared to CD4^+^ T cells from the same individual. Therefore, multiple copies may be more important for studying signalling via the B cell receptor (BCR) when compared with the TCR. Here, in order to avoid variable brightness between littermates, we proceeded to analyse single copy variant mice from founder line N7TD. *In vivo* activation of T cells by intraperitoneal injection of anti-CD3 also drove expression of FT Blue in splenic CD4^+^ and CD8^+^ T cells (Fig. 1F). FT Blue was highly correlated with the activation marker CD69 (Fig. 1G), and occurred in all T cells, demonstrating the utility of Nur77-Tempo mice for tracking TCR signals *in vivo*.

**Figure 2:**
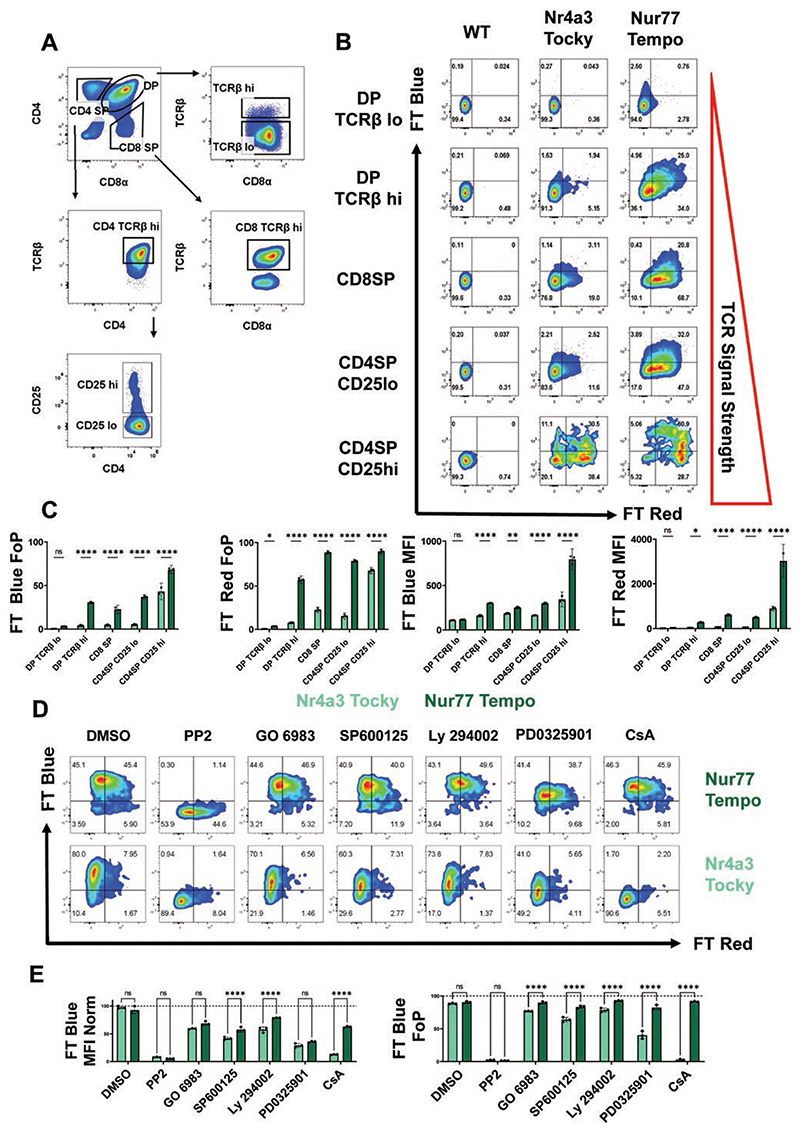
Nur77-Tempo shows increased sensitivity and more graded expression than Nr4a3 Tocky. (A) Gating strategy for subsets of developing thymocytes. (B) Expression of FT Blue and FT Red in thymocyte subsets of *Nr4a3*-Tocky and *Nur77-Tempo* mice. (C) Quantification of (B) displayed as the frequency of parent (FoP; % of indicated subset positive for FT Blue or FT Red) or MFI *n* = 3/group. (D) Expression of FT Blue and FT Red in splenic CD4^+^ T cells following 4-hour *in vitro* stimulation with soluble anti-CD3 in the presence of (0.1% DMSO, 20 μM PP2, 400 nM GO 6983, 10 μM SP600125, 200 nM Ly 294002, 5 μM PD0325901, 1 μM CsA). (E) Quantification of (D) displayed as MFI normalized to MFI of DMSO condition or FoP (% live CD4^+^ T cells positive for FT Blue) *n* = 3/group. Statistical tests are two-way ANOVA, with Sidak’s multiple comparisons test. *P ≤ 0.05, **P ≤ 0.01, ***P ≤ 0.001, ****P ≤ 0.0001.

Our previous work has shown that the expression of Nur77-GFP can be driven by weaker TCR signals than *Nr4a3*-Tocky [19]. We hypothesized that Nur77-Tempo would be better able to detect positive selection signals in the thymus compared to *Nr4a3*-Tocky. Low expression of *Nr4a3*-Tocky in the thymus was observed in all subsets except CD4SP CD25^+^ developing Tregs that have high affinity for self-peptide major-histocompatibility complex (self-pMHC) [25]. Developing Tregs showed high levels of persistent expression as previously described (Fig. 2A–C; Supplementary Fig. 3) [21]. In contrast, high levels of FT Blue and FT Red expression in Nur77-Tempo mice were observed in all TCRβ+ thymic subsets, demonstrating that thymic selection signals occur frequently in both the medulla and cortex. Additionally, the brightness of the reporter was highest in developing Tregs (Fig. 2A–C; Supplementary Fig. 3), with the majority displaying FT Blue demonstrating antigen recognition at the time of analysis. Normalized expression of FT Blue in Nur77-Tempo mice was more sensitive to lower doses of anti-CD3 stimulation *in vitro*, confirming increased sensitivity to TCR signals (Supplementary Fig. 4). These results highlight the sensitivity of Nur77-Tempo to positive selection signals, which are persistent in nature, and graded *Nr4a1* expression in relation to antigen affinity, as has been reported for Nur77-GFP mice [17].

To understand which pathways downstream of the TCR were regulating the expression of FT protein in Nur77-Tempo, we stimulated CD4 T cells in the presence of small molecule inhibitors: PP2 (Src family kinases), GO-6983 {Protein Kinase C [PKC]}, SP600125 {c-Jun N-terminal kinase [JNK]}, Ly-294002 {Phosphoinositide-3-kinase [PI3K]}, PD0325901 (ERK), and CsA {Calcineurin [NFAT Pathway]; Fig. 2D and E}.

*Nr4a3*-Tocky showed greater sensitivity to CsA than Nur77-Tempo, as previously reported for Nur77-GFP mice [19]. Both reporters showed a similar decrease in normalized MFI in response to all inhibitors, indicating that FT protein expression in both Nur77-Tempo and *Nr4a3*-Tocky is partially regulated by all pathways tested. Despite reductions in MFI, the Nur77-Tempo reporter showed limited reductions in the proportions FT Blue^+^. In comparison, *Nr4a3*-Tocky showed more significant reductions, this contrast was best exemplified by inhibition of the ERK pathway with PD0325901 (Fig. 2C). Therefore, whilst reductions in MFI of FT protein in *Nr4a3*-Tocky mice were largely driven by a binary loss in the proportion of cells crossing the threshold to express detectable FT protein, reductions in Nur77-Tempo driven FT protein MFI were driven by a graded reduction in brightness in those cells already making the reporter.

**Figure 3:**
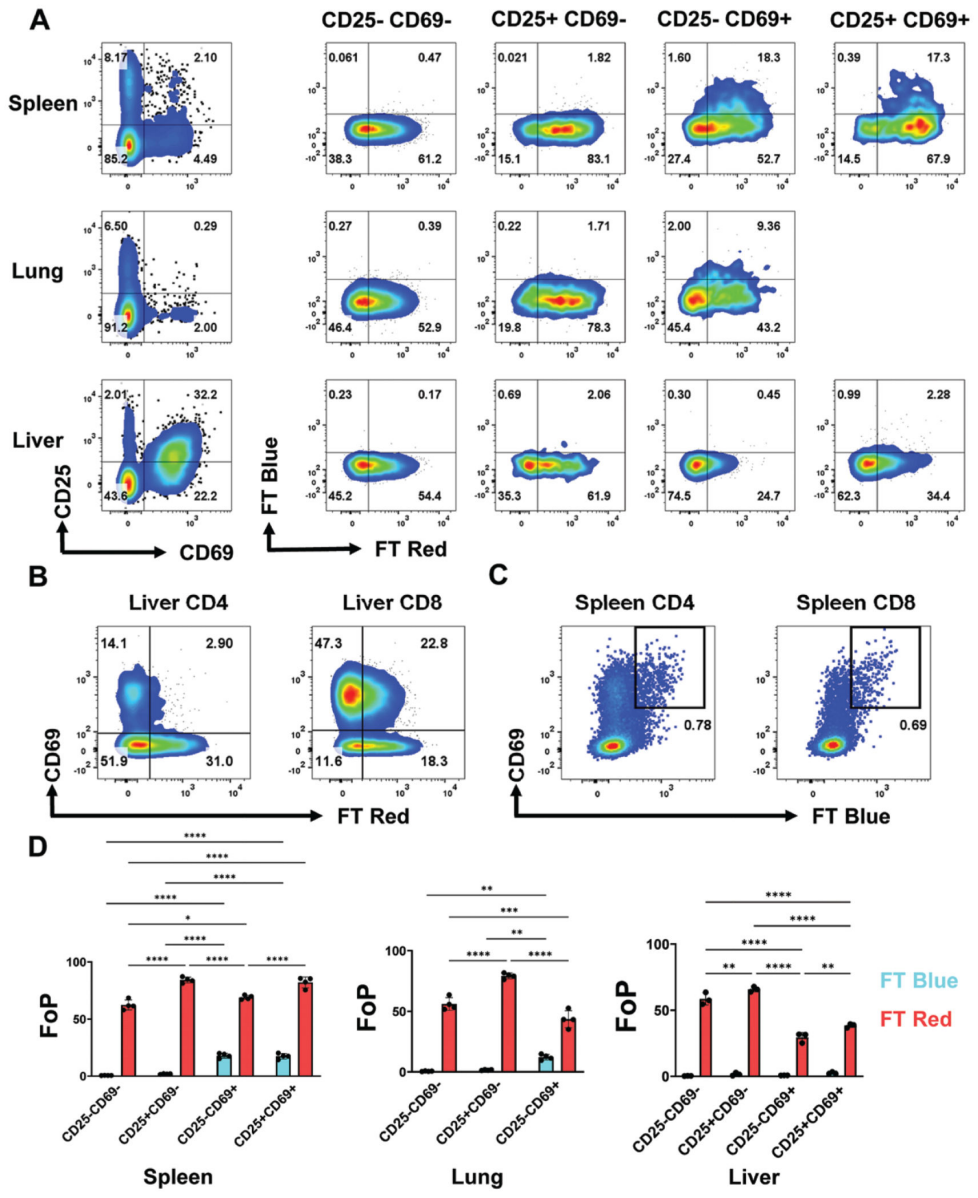
Tracking of steady state antigen recognition in lymphoid and peripheral tissue. (A) Gating strategy for CD4 T cell subsets based on CD69 and CD25 expression, pre-gated on live CD4^+^ T cells. Expression of FT Blue and FT Red in indicated subsets from spleen, lung, and liver (tissue preparations detailed in methods). (B) Expression of FT Red and CD69 in liver CD4 and CD8 T cells. (C) Expression of CD69 and FT Blue in spleen CD4 and CD8 T cells. (D) Quantification of (A) displayed as FoP (% of subset positive for FT Blue and FT Red) *n* = 3. Statistical tests are two-way ANOVA, with Sidak’s multiple comparisons test. *P ≤ 0.05, **P ≤ 0.01, ***P ≤ 0.001, ****P ≤ 0.0001.

Due to the ability of Nur77-Tempo to detect low-affinity TCR signals in thymocytes, we hypothesized that we could detect basal TCR signalling in steady state mature T cells. The majority of splenic CD4^+^ T cells expressed FT Red (Fig. 3A and D), likely driven by a history of low strength self-antigen recognition that is necessary for T cell survival and priming [26]. CD4^+^ CD25^+^ populations are enriched for strongly autoreactive FoxP3+ Tregs [25], accordingly, CD25 expression was associated with higher expression of FT Red (Fig. 3A and D). The CD69^+^ population is enriched for T cells retained in the lymphoid environment in response to priming signals, likely from a foreign antigen or CD69 expression in response to tonic TCR signalling. FT Blue expression was restricted to CD69^+^ CD4^+^ and CD8^+^ T cells (Fig. 3A and C; Supplementary Fig. 5), indicating that priming signals are more frequent in nature than those tonic signals that do not drive expression of CD69. However, we cannot exclude the possibility that CD69^+^ cells have not received a priming signal in response to foreign antigen and instead tonic signalling causes expression of CD69 that is very transient, such that only ~5% of splenic CD4 T cells are CD69^+^ at any given time.

We sought to understand the role of antigen recognition in the residency of T cells within peripheral tissues. Although low levels of FT Blue expression were observed in the lung, the majority of CD69^+^ CD4 and CD8 T cells in the lung and liver were FT Blue^-^-(Fig. 3A and D; Supplementary Fig. 5). Furthermore, in both the lung and liver, CD69 expression was associated with a decrease in expression of FT Red (Fig. 3A, B, D). Therefore, tissue residency is not associated with recent peripheral antigen recognition, but in fact, a loss of low frequency self-antigen recognition that is observed in lymphoid tissue. Intraepithelial lymphocytes (IELs) from the small intestine showed high expression of CD69 in all subsets, as expected (Supplementary Fig. 6) [27]. TCRβ^+^ CD8αβ^+^ IELs that likely arrived from the circulation mirrored our observations of liver T cells, showing less FT Red expression than splenic CD8^+^ T cells. In contrast, CD8αα^+^ IELs that were either TCRβ^+^ or TCRγδ^+^ were >80% FT Red^+^, and the TCRγδ^+^ population were ~5% FT Blue^+^ (Supplementary Fig. 6). These results suggest antigen recognition within the epithelial layer is likely more frequent for CD8αα^+^, than for CD8αβ^+^, IELs.

## Discussion

Here, we have validated the use of Nur77-Tempo mice for investigating TCR signalling dynamics. Although extensively reported elsewhere [17, 18], we have not used this model to directly test the antigen specificity of *Nr4a1* expression, which future research should consider when utilizing this tool. We have compared the model to *Nr4a3*-Tocky, which has been used previously to investigate NFAT-driven TCR signalling dynamics, identifying key differences. The Nur77-Tempo model has certain advantages over *Nr4a3*-Tocky, such as more graded expression patterns in response to TCR signal strength, and detection of weak/lower affinity TCR signals. We have previously used *Nr4a3*-Tocky to study changes to TCR signal strength, demonstrating subtle changes to the brightness of FT Blue in response to co-inhibitory receptor blockade (Elliot et al., 2021). However, our results here show that the graded nature of Nur77-Tempo may be more sensitive for similar future investigations into the dynamics of TCR signal strength.

FT protein expression in Nur77-Tempo mice was detectable in all TCRβ thymocytes, whilst expression in *Nr4a3*-Tocky is restricted to developing Tregs. Nur77-Tempo is therefore a more powerful tool for studying TCR signalling dynamics during early thymic events such as positive selection. A caveat to the increased sensitivity of Nur77-Tempo is high baseline expression in lymphoid tissue, with ~70% of CD4 T cells expressing FT Red in the spleen. This indicates that most splenic T cells receive tonic signals that are strong enough to induce the expression of *Nr4a1* but not *Nr4a3*. Therefore, if future studies aim to study TCR signalling dynamics where a binary read-out is acceptable but high background is not, *Nr4a3*-Tocky is clearly advantageous.

The function of CD69 in lymphoid tissue is to retain primed T cells. Accordingly, the splenic CD69^+^ T cell population was enriched for FT Blue^+^ cells, indicative of recent TCR signalling events. In peripheral tissues, the role of CD69 is to retain resident T cells. Antigen-presenting cells (APCs) in peripheral organs can be of both hematopoietic and epithelial lineage [28], and although antigen recognition does occur in peripheral tissues, it is unclear whether it is required for residency programs. Whilst CD69^+^ T cells were rare in the lung, a large CD69^+^ population was observed in the liver. In contrast to the spleen, CD69^+^ T cells in the liver were almost entirely FT Blue^-^, indicating that CD69 expression was not driven by recent antigen recognition, but the liver microenvironment. Furthermore, we observed a relative loss of FT Red expression in liver CD69^+^ T cells, suggesting that resident T cells no longer receive frequent self-antigen TCR signals and that their survival is maintained by the tissue microenvironment. Interestingly, this reduction in FT Red expression was observed even in the CD25^+^ liver CD4 T cells, suggesting that autoreactive Tregs do not require continued self-antigen recognition to maintain CD25 expression. We observed high levels of FT Red expression in CD8αα^+^ IELs, suggesting constitutive TCR ligation within the epithelial layer. It is possible that this is due to the recognition of microbial antigens, which are abundant in the intestine or, in the case of γδ T cells, binding to butyrophillin-like molecules expressed by intestinal epithelial cells [29].

In summary, we have generated a new transgenic reporter of antigen receptor signalling that combines the time-dependent maturation of FT protein and the sensitive and graded expression patterns of *Nr4a1*/Nur77. We have used this transgenic mouse line to track steady state TCR signalling, revealing the persistent nature of thymic positive selection signals and the relative paucity of tonic self-antigen recognition in T cells residing in peripheral tissue.

## Supplementary Material

Supplementary Figures

## Data Availability

The authors declare that data will be made available upon reasonable request. For use of the *Nr4a3*-Tocky line please contact Dr Masahiro Ono, (m.ono@imperial.ac.uk) Imperial College London.

## Abbreviations

BCR: B cell receptor
ERK: extracellular signal related-kinase
FT: Fluorescent Timer
GFP: Green Fluorescent Protein
ITK: Interleukin-2-Inducible Kinase
MFI: median fluorescent intensity
MHC: Major Histocompatibility Complex
NF-κB: Nuclear Factor Kappa B
NFAT: Nuclear factor of activated T cells
S1PR_1_: sphingosine-1-phosphate receptor 1
TCR: T cell receptor
Tempo: Timer rapidly expressed in lymphocytes
Tocky: Timer of cell kinetics and activity
