# Supplementary material for: Nur77-Tempo mice reveal T cell steady state antigen recognition
Source: Discov Immunol. 2022 Nov 21;1(1):kyac009. doi: 10.1093/discim/kyac009 (PMC7614040; doi:10.1093/discim/kyac009)
Supplement: kyac009_suppl_Supplementary_Figures [file kyac009_suppl_Supplementary_Figures.pdf]

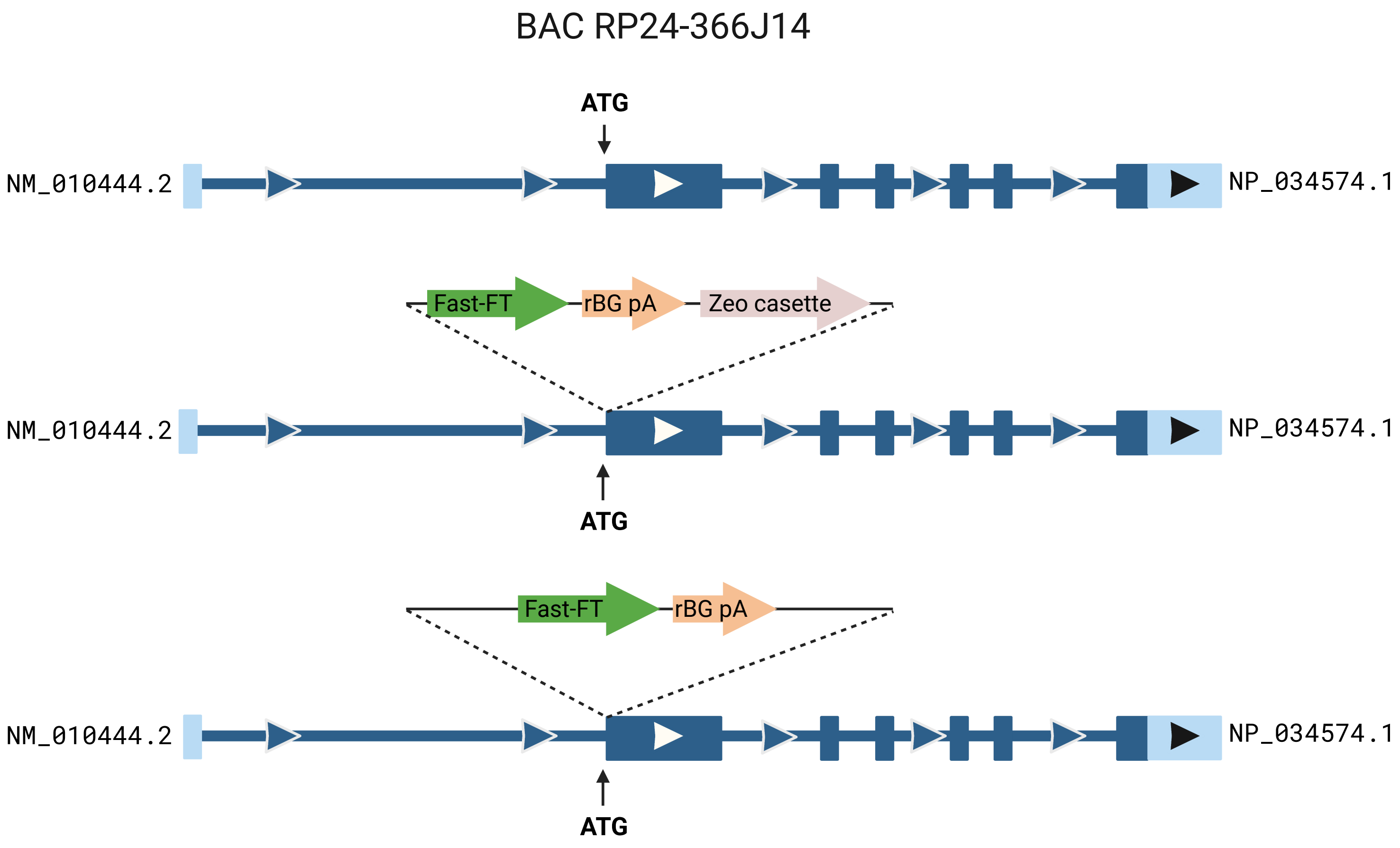

Supplementary Figure 1. *Nur77*-Tempo BAC targeting approach

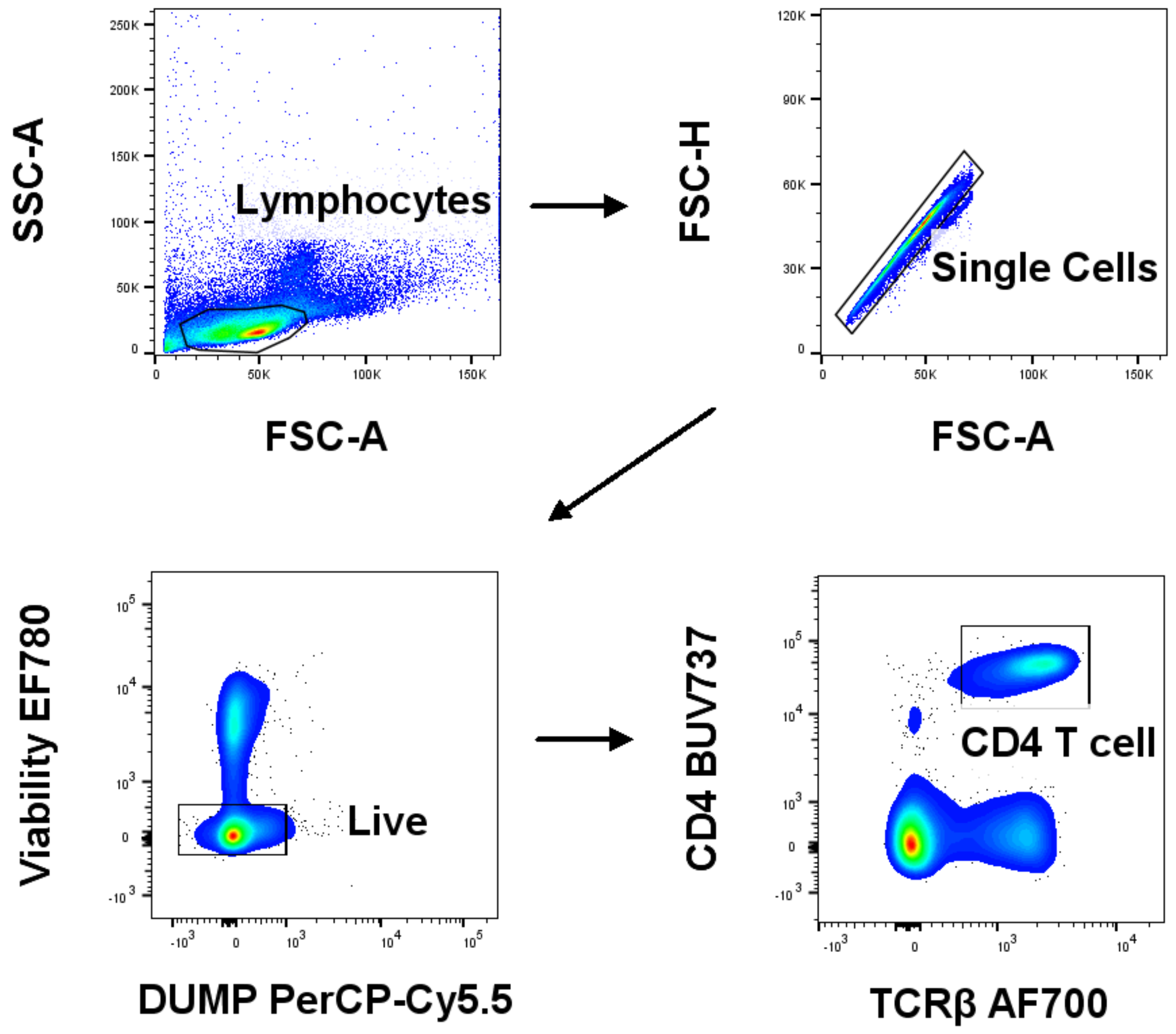

Supplementary Figure 2. Gating strategy for CD4⁺ T cells for analysis of FT Blue and FT Red expression by flow cytometry.

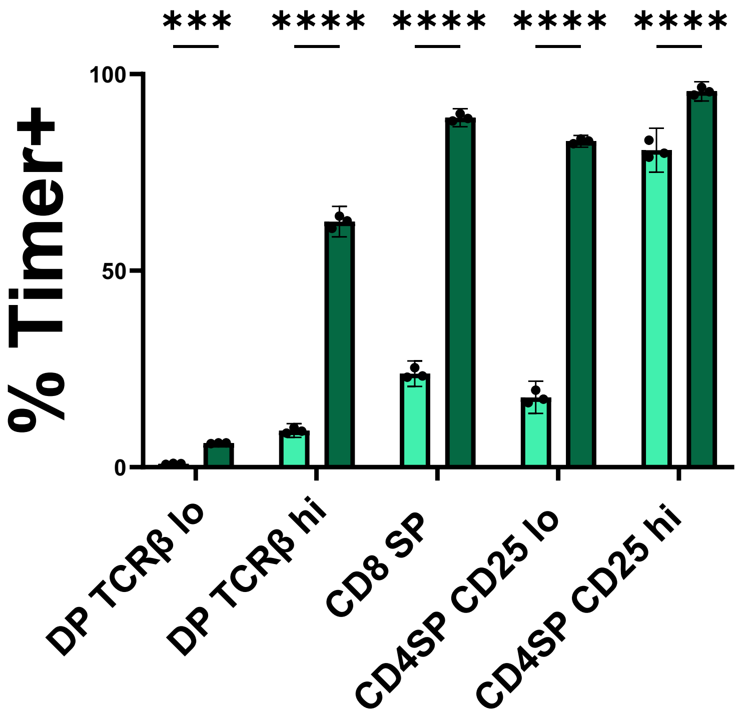

Supplementary Figure 3. % Timer⁺ cells of different thymic subsets in Nur77-Tempo and *Nr4a3*-Tocky mice. Relates to figure 2C. n = 3. Light green = *Nr4a3*-Tocky, dark green = *Nur77*-Tempo. Statistical test by two-way ANOVA with Sidak's multiple comparisons test. * P ≤ 0.05 **P ≤0 .01 ***P ≤ 0.001 ****P ≤ 0.0001.

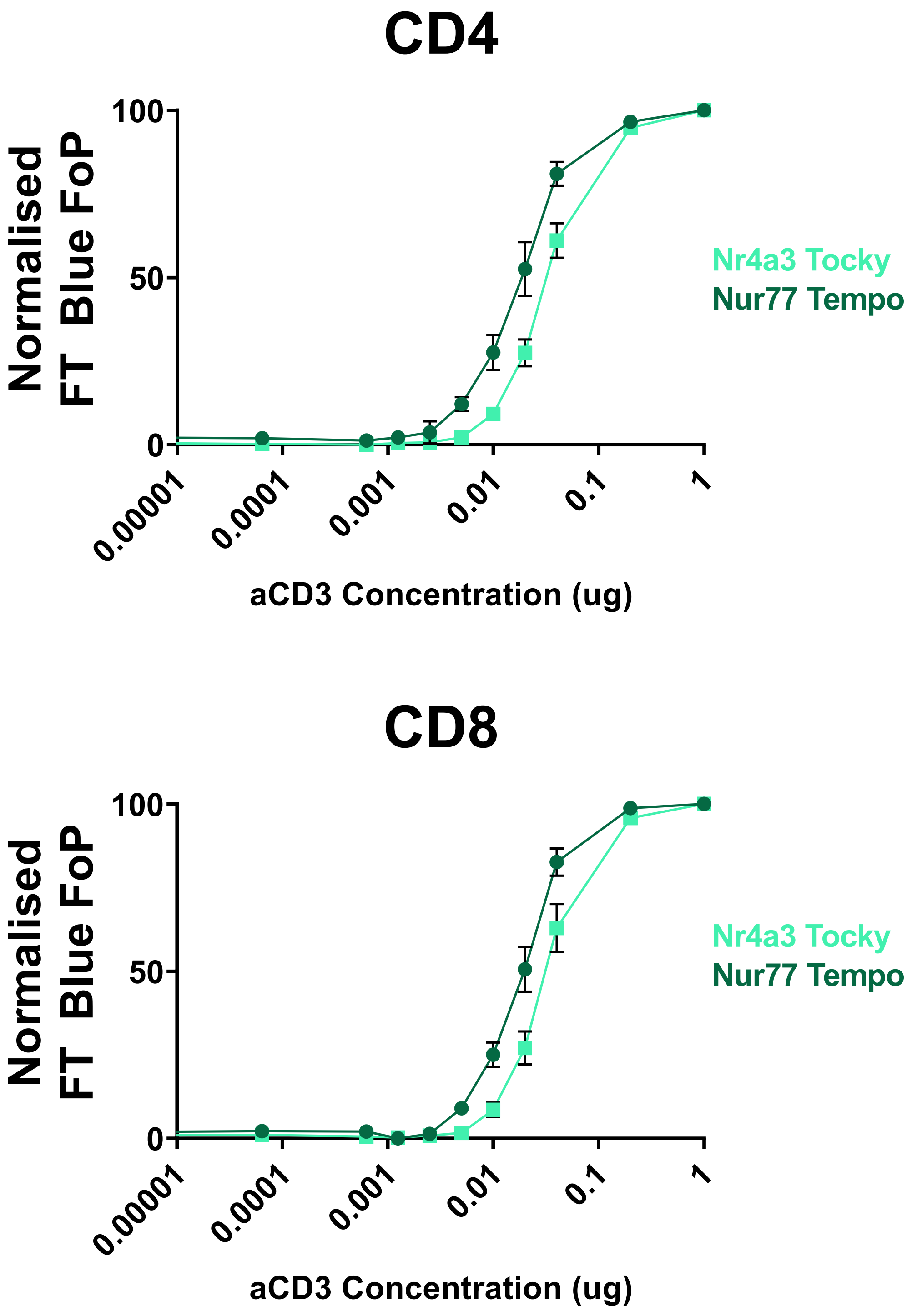

Supplementary Figure 4. Expression of FT Blue in splenic CD4⁺ and CD8⁺ T cells following 4 hour in vitro stimulation with dose titration of soluble anti-CD3. Data is displayed as frequency of parent (% of TCRβ⁺CD4/CD8⁺ cells FT Blue⁺) (FoP) normalised by (((FoP – minimum FoP) / maximum FoP) * 100). N=3, bars represent mean±SEM

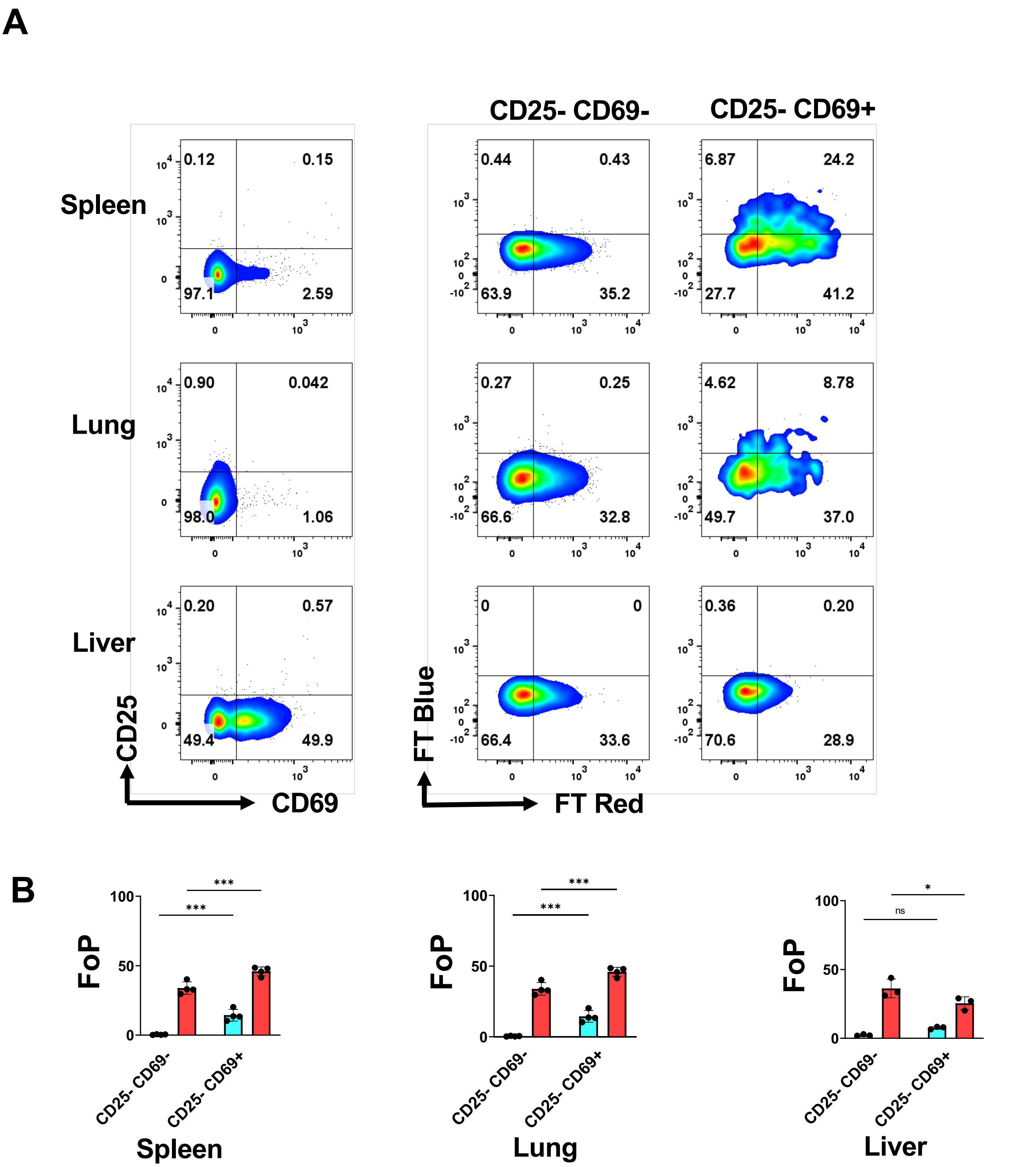

Supplementary Figure 5. Tracking of steady state antigen recognition in lymphoid and peripheral tissue in Nur77-Tempo mice. Relates to figure 3A. A) Gating strategy for $CD8^+$ T cell subsets based on CD69 and CD25 expression, pre-gated on live $CD8^+$ T cells. Expression of FT Blue and FT Red in indicated subsets from spleen, lung, and liver (tissue preparations detailed in methods). B) Quantification of (A) displayed as FoP (% of subset positive for FT Blue and FT Red) n = 3. Statistical tests are two way ANOVA with Sidak's multiple comparisons test. * $P \leq 0.05$ **$P \leq 0.01$ ***$P \leq 0.001$ ****$P \leq 0.0001$.

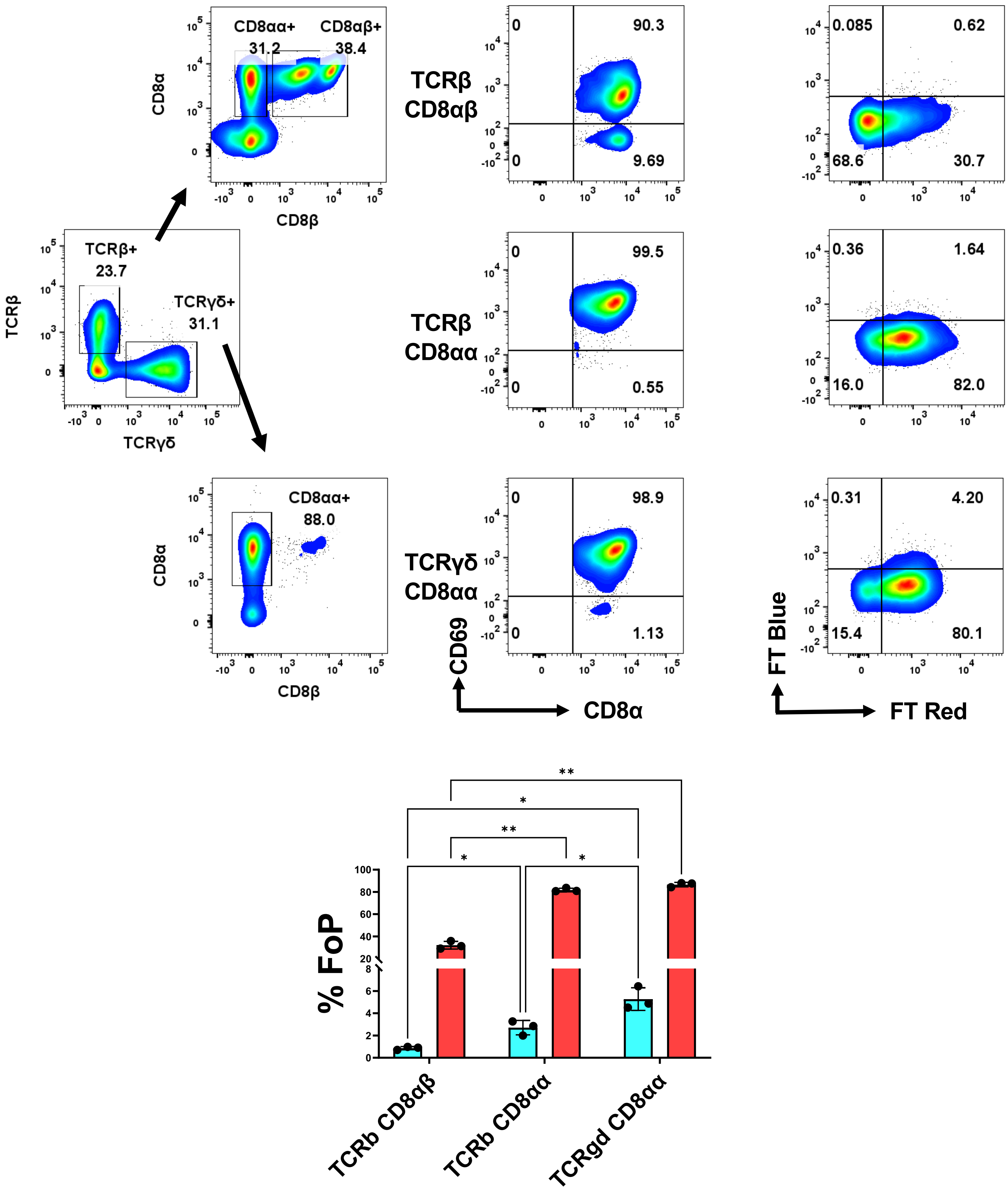

Supplementary Figure 6. Tracking of steady state antigen recognition in epithelial layer of the small intestine in Nur77-Tempo mice. A) Gating strategy for T cell subsets based on TCRβ, TCRγδ, CD8α, and CD8β expression. Pre-gated on live singlets. Expression of FT Blue and FT Red in indicated subsets (tissue preparations detailed in methods). B) Quantification of (A) displayed as FoP (% of subset positive for FT Blue and FT Red) n = 3. Statistical testing by two-way ANOVA with Sidak's multiple comparisons test. * $P \leq 0.05$ **$P \leq 0.01$ ***$P \leq 0.001$ ****$P \leq 0.0001$.
